# Systematic Analysis of the Functions of Lysine Acetylation in the Regulation of Tat Activity

**DOI:** 10.1371/journal.pone.0067186

**Published:** 2013-06-27

**Authors:** Minghao He, Linlin Zhang, Xincheng Wang, Lihong Huo, Lei Sun, Chengye Feng, Xutian Jing, Danyao Du, Huabin Liang, Min Liu, Zhangyong Hong, Jun Zhou

**Affiliations:** 1 Center for AIDS Research, College of Life Sciences, Nankai University, Tianjin, China; 2 Tianjin Key Laboratory of Medical Epigenetics, Tianjin Medical University, Tianjin, China; George Mason University, United States of America

## Abstract

The Tat protein of HIV-1 has several well-known properties, such as nucleocytoplasmic trafficking, transactivation of transcription, interaction with tubulin, regulation of mitotic progression, and induction of apoptosis. Previous studies have identified a couple of lysine residues in Tat that are essential for its functions. In order to analyze the functions of all the lysine residues in Tat, we mutated them individually to alanine, glutamine, and arginine. Through systematic analysis of the lysine mutants, we discovered several previously unidentified characteristics of Tat. We found that lysine acetylation could modulate the subcellular localization of Tat, in addition to the regulation of its transactivation activity. Our data also revealed that lysine mutations had distinct effects on microtubule assembly and Tat binding to bromodomain proteins. By correlation analysis, we further found that the effects of Tat on apoptosis and mitotic progression were not entirely attributed to its effect on microtubule assembly. Our findings suggest that Tat may regulate diverse cellular activities through binding to different proteins and that the acetylation of distinct lysine residues in Tat may modulate its interaction with various partners.

## Introduction

The Tat protein plays an essential role in the life cycle of HIV-1. Tat has many biological functions, such as transactivation of transcription, regulation of mitotic progression, and induction of apoptosis [1]–[4]. Like the activators of many other viruses, Tat regulates HIV-1 gene transcription primarily through association with host cellular factors. This process provides a positive feedback cycle, allowing HIV-1 to have an explosive response to defeat the body's response. Tat can also act directly as a toxin to induce apoptosis in uninfected “bystander” cells [1]–[3], [5]. In addition, Tat binds to tubulin, stimulates microtubule assembly, and causes the formation of abnormally stable microtubules, which contributes to the progressive loss of T lymphocytes [6], [7]. Over the past decades, intensive efforts have been spent on studying Tat functions [8]. However, the mechanism of action of this small protein remains incompletely understood.

HIV-1 Tat is encoded by two exons. The first exon encodes amino acids 1–72 in most HIV-1 strains, with amino acids 1–13 harboring amphipathic characteristics, 22–37 containing several cysteines to form intra-molecular disulfide bond, and 49–72 rendering its binding to the transactivation-responsive RNA (TAR) [9], [10]. In addition, the region of amino acids 1–48 acts as an activation domain that mediates the interaction of Tat with cellular factors, such as Cyclin T1 [11]. The second exon encodes amino acids 73–101 of Tat and is important for its transactivation activity, interaction with tubulin, and viral replication, albeit the precise molecular mechanisms remain to be investigated [12]–[14]. Most of the previous studies on Tat have been performed on truncated forms of Tat, such as the 72- or 86-amino acid forms. Although the truncated forms of Tat contain essential elements for viral replication, Tat in most HIV-1 strains has 101 amino acids. Therefore, the 101-amino acid form of Tat was used in the present study.

Tat is known to undergo post-translational modifications, such as acetylation, ubiquitination, and phosphorylation, which play a critical role in the regulation of diverse Tat functions [15]. In particular, Tat acetylation at lysine-28 (K28) and lysine-50 (K50) could regulate its transactivation activity [16]–[20]. Acetylation at K50 and K51 could regulate the interaction of Tat with Brg1, Brm, and bromodomain proteins such as the histone acetyltransferase Gcn5 and p300/CBP-associated factor (PCAF) [21]–[24]. In addition, Tat acetylation at K28 could modulate its actions on microtubule dynamics and apoptosis in T lymphocytes [7] **.** However, it remains largely elusive whether other lysine residues undergo acetylation and whether their acetylation regulates Tat functions. In the present study, all the 14 lysine residues in Tat were mutated individually to alanine (to neutralize electric charge and block acetylation), glutamine (to neutralize electric charge and mimic acetylation), and arginine (to preserve electric charge and block acetylation). Through systematic analysis of the lysine mutants, we discovered several previously unidentified characteristics of Tat.

## Materials and Methods

### Antibodies and Plasmids

Mouse anti-α-tubulin monoclonal antibody was purchased from Sigma-Aldrich, mouse anti-GFP monoclonal antibody was from Roche, and rabbit anti-Gcn5 polyclonal and mouse anti-PCAF monoclonal antibodies were from Santa Cruz Biotechnology. Horseradish peroxidase-conjugated secondary antibodies were obtained from Amersham Biosciences. Anti-GFP antibody-conjugated agarose beads were from MBL. The mammalian expression plasmid for GFP-Tat was constructed by insertion of HIV-1 Tat cDNA into the pEGFPC1 vector, and various lysine mutants were generated by site-directed mutagenesis. The HIV-1 long terminal repeat (LTR)-driven luciferase plasmid has been described previously [17].

### Cell Culture and Transfection

293T and Jurkat cells were cultured in Dulbecco’s modified Eagle’s medium supplemented with 10% fetal bovine serum at 37°C in a humidified atmosphere with 5% CO_2_. Plasmids were transfected to 293T cells by using the polyethyleneimine reagent (Sigma-Aldrich) and transfected to Jurkat cells by using the DMRIE-C reagent (Invitrogen).

### Fluorescence Microscopy

Cells grown on glass coverslips were fixed with 4% paraformaldehyde for 30 minutes at room temperature and blocked with 2% bovine serum albumin in PBS. Coverslips were mounted with 90% glycerol in phosphate-buffered saline and examined with an Axio Observer A1 fluorescence microscope (Carl Zeiss).

### Luciferase Reporter Assay

Cells were transfected with the HIV-1 LTR-driven luciferase plasmid and a β-galactosidase-expressing plasmid. The luciferase activity was measured using an FB12 luminometer (Berthold Detection Systems) and normalized to β-galactosidase activity. To measure the transactivation activity of Tat, cells were transfected with the GFP-Tat expression plasmid or empty vector in addition to the above plasmids, and the extent of transactivation was determined.

### Preparation of Soluble Tubulin

Cells were lysed in the PEMG buffer (100 mM PIPES, 1 mM EGTA, 1 mM MgSO_4_, 1 mM GTP, pH 6.8) containing 0.1% Triton X-100 and 4 M glycerol, and the supernatant was then collected as described [25].

### Immunoblotting and Immunoprecipitation

Protein samples were resolved by SDS-PAGE and transferred onto polyvinylidene difluoride membranes (Millipore). The membranes were incubated first with mouse anti-α-tubulin monoclonal antibody and then with horseradish peroxidase-conjugated anti-mouse secondary antibody. Specific proteins were visualized with enhanced chemiluminescence detection reagent (Millipore). For immunoprecipitation, cell lysate was incubated with anti-GFP antibody-conjugated agarose beads at 4°C for 2 hours, and the precipitated proteins were then examined by immunoblotting.

### Analysis of Mitotic Arrest and Apoptosis

Cells grown on coverslips were transfected with various GFP-Tat plasmids. The percentage of mitotic cells was then quantified 48 hours later by fluorescence microscopic analysis of nuclear morphology, and the percentage of apoptotic cells was quantified 96 hours later by fluorescence microscopic analysis of nuclear morphology.

## Results

### Tat Lysine Mutants are Expressed at Different Levels in Cells

**Figure 1:**
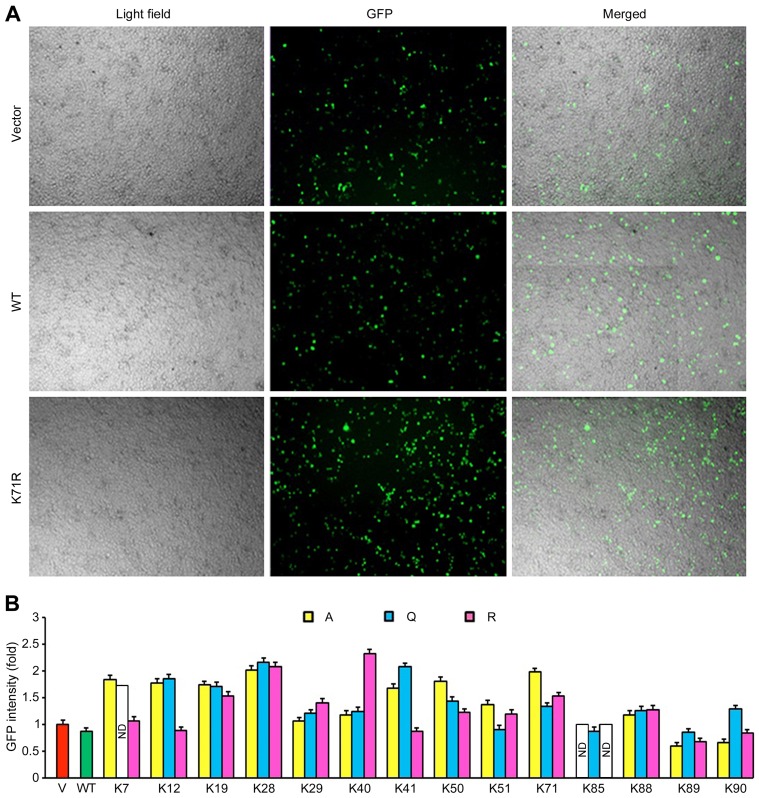
Tat lysine mutants are expressed at different levels in cells. (A) 293T cells were transfected with various GFP-Tat plasmids or the empty vector, and the fluorescence of GFP was examined by fluorescence microscopy 24 hours after transfection. (B) Experiments were performed as in panel A, and the expression levels of the GFP fusion proteins were measured via the ImageJ software and normalized to the vector group. WT, wild-type; ND, not determined.

To better understand the functions of Tat acetylation, we constructed a series of Tat mutant plasmids using the pEGFPC1 vector. All the 14 lysine residues in Tat were mutated one by one to alanine, glutamine, and arginine. In these mutants, GFP was used as a reporter for the expression of GFP-Tat fusion proteins. To check the expression of the mutants, 293T cells were transfected with the series of pEGFPC1-Tat plasmids, and the fluorescence of GFP was examined by fluorescence microscopy 24 hours after transfection (Fig. 1A). The transfection efficiency was calculated by comparing the number of GFP-positive cells with the number of total cells in the same field. We found that the transfection efficiency of GFP alone, GFP-Tat wild-type, and GFP-Tat mutants was in the range of 10–30%. The expression levels of the GFP fusion proteins were then measured with the ImageJ software, and the results showed that these proteins were expressed at different levels in cells (Fig. 1B).

### Lysine Mutations Modulate the Subcellular Localization of Tat

**Figure 2:**
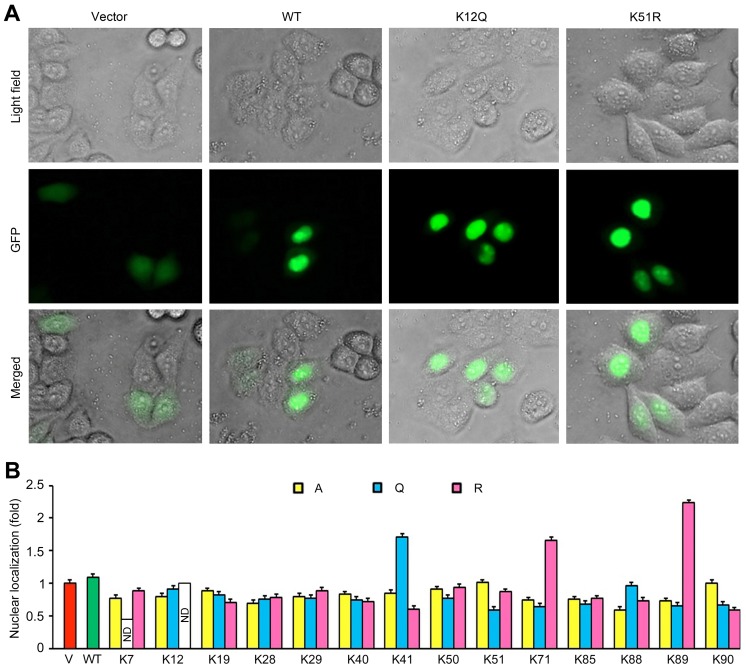
Lysine mutations modulate the subcellular localization of Tat. (A) 293T cells were transfected with different GFP-Tat plasmids or the empty vector, and the subcellular localization of GFP alone or GFP-Tat fusion proteins was examined by fluorescence microscopy. (B) Experiments were performed as in panel A, and the percentage of nuclear Tat was quantified and normalized to the vector group. WT, wild-type; ND, not determined.

Tat is known to undergo nucleocytoplasmic trafficking and localizes primarily in the nuclei with a small proportion in the cytoplasm, and this localization pattern is critical for diverse Tat functions [26]. We thus examined the subcellular localization of Tat in cells by fluorescence microscopy and quantified with ImageJ. Wild-type Tat and the majority of Tat mutants displayed a clear nuclear distribution pattern and the fluorescence of GFP was mainly observed in the nuclei (Fig. 2A). In contrast, the K90R mutation significantly impaired the nuclear localization of Tat (Fig. 2A). The nuclear localization of Tat was then quantified by measuring the ratio of green fluorescence intensity inside the nucleus to that in the whole cell. Compared with wild-type Tat, the K41Q, K71R and K89R mutations significantly increased the accumulation of Tat in the nucleus, whereas the K41R, K51Q, K88A, and K90R mutations decreased the nuclear localization of Tat (Fig. 2B). These data thus demonstrate that Tat localizes predominantly in the nucleus and that the mutation of lysine residues in Tat could influence its subcellular localization.

### Tat Transactivation Activity is Exquisitely Regulated by Lysine Acetylation

**Figure 3:**
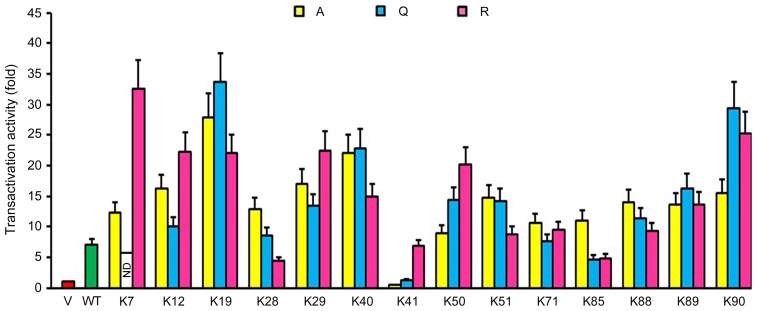
Tat transactivation activity is exquisitely regulated by lysine acetylation. 293T cells were transfected with an HIV-1 LTR-driven luciferase plasmid, a β-galactosidase-expressing plasmid, and various GFP-Tat plasmids or the empty vector. Luciferase activity was then measured and normalized to β-galactosidase activity, and the data were subsequently normalized to the vector group. WT, wild-type; ND, not determined.

Tat acetylation at K28 and K50 is critical for its transactivation activity towards HIV-1 transcription [16]–[18]. To investigate whether the acetylation of other lysine residues affects the transactivation activity of Tat, we performed luciferase reporter assay using an HIV-1 LTR-driven luciferase plasmid and a β-galactosidase-expressing plasmid. The β-galactosidase activity was measured as an internal control to normalize for the difference in transfection efficiency. Consistent with previous findings [16]–[18], the transactivation activity of Tat was greatly affected by mutations in K28 and K50 residues. Compared with the wild-type control, the K7R, K12R, K19A, K19Q, K19R, K29R, K40A, K40Q, K50R, K90Q, and K90R mutations remarkably increased Tat transactivation activity. In contrast, the K28R, K41A, K41Q, K85Q, and K85R mutations exerted negative effects (Fig. 3). These data suggest that Tat transactivation activity is exquisitely regulated by lysine acetylation.

### Tat Lysine Mutations have Distinct Effects on Microtubule Assembly

**Figure 4:**
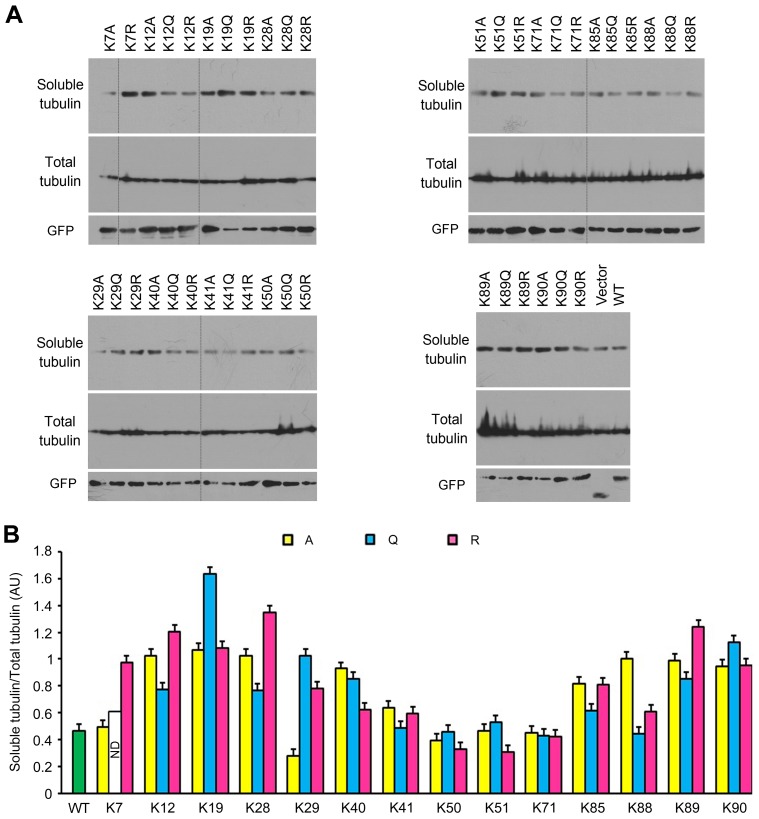
Tat lysine mutations have distinct effects on microtubule assembly. (A) 293T cells were transfected with different GFP-Tat plasmids or the empty vector. Cell extracts that contain soluble tubulin or total tubulin were prepared 24 hours after transfection. Immunoblotting was then performed with anti-α-tubulin antibody to examine the levels of soluble tubulin and total tubulin, and with anti-GFP antibody to examine the levels of GFP-tagged proteins. (B) Experiments were performed as in panel A, and the percentage of soluble tubulin was quantified. WT, wild-type; ND, not determined.

Acetylation of K28 has been demonstrated to regulate the effect of Tat on microtubule assembly [7]. We thus asked whether Tat acetylation at other lysine residues affects its action on microtubule assembly. 293T cells were transfected with the mutants individually. Cell extracts that contain soluble tubulin or total tubulin were prepared 24 hours after transfection. Immunoblotting was then performed with anti-α-tubulin antibody to examine the percentage of soluble tubulin among total tubulin. In each group, cells transfected with the pEGFPC1 vector was used as control (Fig. 4A). Compared with the wild-type Tat group, we found that the K29A, K50R, and K51R mutations decreased the proportion of soluble tubulin. However, the majority of other mutations increased the percentage of soluble tubulin. We also found that there was no significant difference between the effects of wild-type Tat and the vector, indicating a weak effect of wild-type Tat on microtubule assembly in 293T cells (Fig. 4B). These results thus reveal that Tat lysine mutations have distinct effects on microtubule assembly.

### The Effect of Tat on Mitotic Progression is not Attributed to its Activity Towards Microtubule Assembly

**Figure 5:**
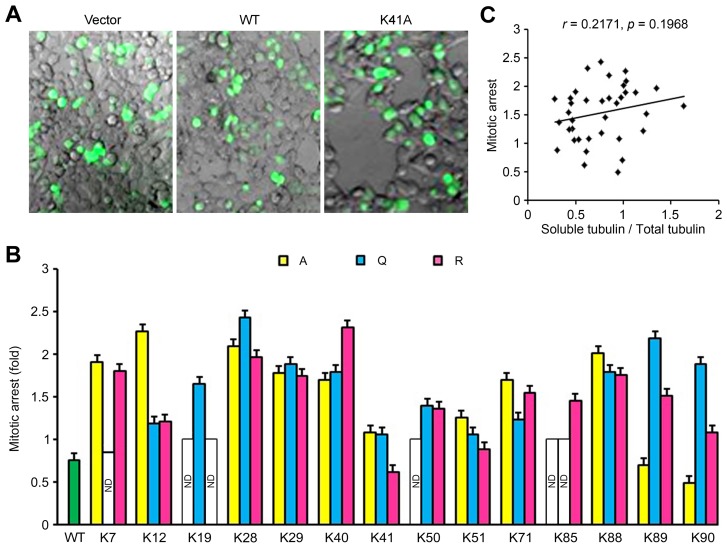
The effect of Tat on mitotic progression is not attributed to its activity towards microtubule assembly. (A) 293T cells were transfected with various GFP-Tat plasmids or the empty vector for 48 hours, and mitotic cells were examined by fluorescence microscopic analysis of nuclear morphology. (B) Experiments were performed as in panel A, and the rate of mitotic arrest was quantified. (C) Data were from Fig. 4B and Fig. 5B, and the correlation between mitotic arrest and the percentage of soluble tubulin was analyzed. WT, wild-type; ND, not determined.

Regulation of mitotic progression is another property of Tat [1]–[3]. To examine whether lysine mutations modulate the activity of Tat to regulate mitosis, cells were transfected with various GFP-Tat plasmids for 48 hours, and the percentage of mitotic cells was quantified by fluorescence microscopic analysis of nuclear morphology (Fig. 5A). Compared with the wild-type Tat group, the K41R and K90A mutations appeared to inhibit the activity of Tat to perturb mitosis. In contrast, most of the other mutations dramatically promoted Tat-induced mitotic arrest (Fig. 5B). To investigate whether the effects of Tat lysine mutations on mitotic arrest result from their effects on microtubule assembly, we analyzed the potential association between mitotic arrest and the percentage of soluble tubulin. By correlation analysis, we found that there was no apparent correlation between these two functions of Tat (Fig. 5C). These findings suggest that the effect of Tat on mitotic progression is not attributed to its activity towards microtubule assembly.

### Tat-induced Apoptosis does not Entirely Result from its Activity Towards Microtubule Assembly

**Figure 6:**
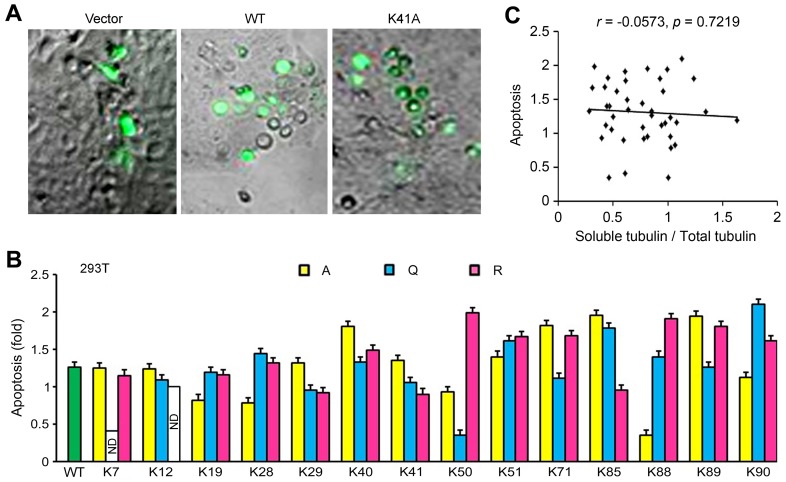
Tat-induced apoptosis does not entirely result from its activity towards microtubule assembly. (A) 293T cells were transfected with various GFP-Tat plasmids or the empty vector for 96 hours, and apoptotic cells were examined by fluorescence microscopic analysis of nuclear morphology. (B) Experiments were performed as in panel A, and the percentage of apoptotic cells was quantified. (C) Data were from Fig. 4B and Fig. 6B, and the correlation between apoptosis and the percentage of soluble tubulin was analyzed. WT, wild-type; ND, not determined.

Tat acetylation at K28 has been reported to regulate the activity of Tat to induce apoptosis [7]. Therefore, we asked whether Tat acetylation at other lysine residues affects Tat-induced apoptosis. To test this, the percentage of apoptosis was analyzed by fluorescence microscopic analysis of nuclear morphology in 293T cells transfected with various Tat plasmids for 96 hours (Fig. 6A). In agreement with previous results [7], wild-type Tat could efficiently induce apoptosis, and this activity was affected by mutations in the K28 residue (Fig. 6B). Interestingly, the K50Q and K88A mutations significantly inhibited the activity of Tat to induce apoptosis, whereas many other mutations such as K40A, 50R, K71A, K85A, K88R, K89A, and K90Q promoted the ability of Tat to induce apoptosis (Fig. 6B). We also analyzed the potential association between Tat-induced apoptosis and the percentage of soluble tubulin. Correlation analysis revealed that that there was no apparent correlation between these two functions of Tat (Fig. 6C), suggesting that Tat-induced apoptosis does not entirely result from its activity towards microtubule assembly.

**Figure 7:**
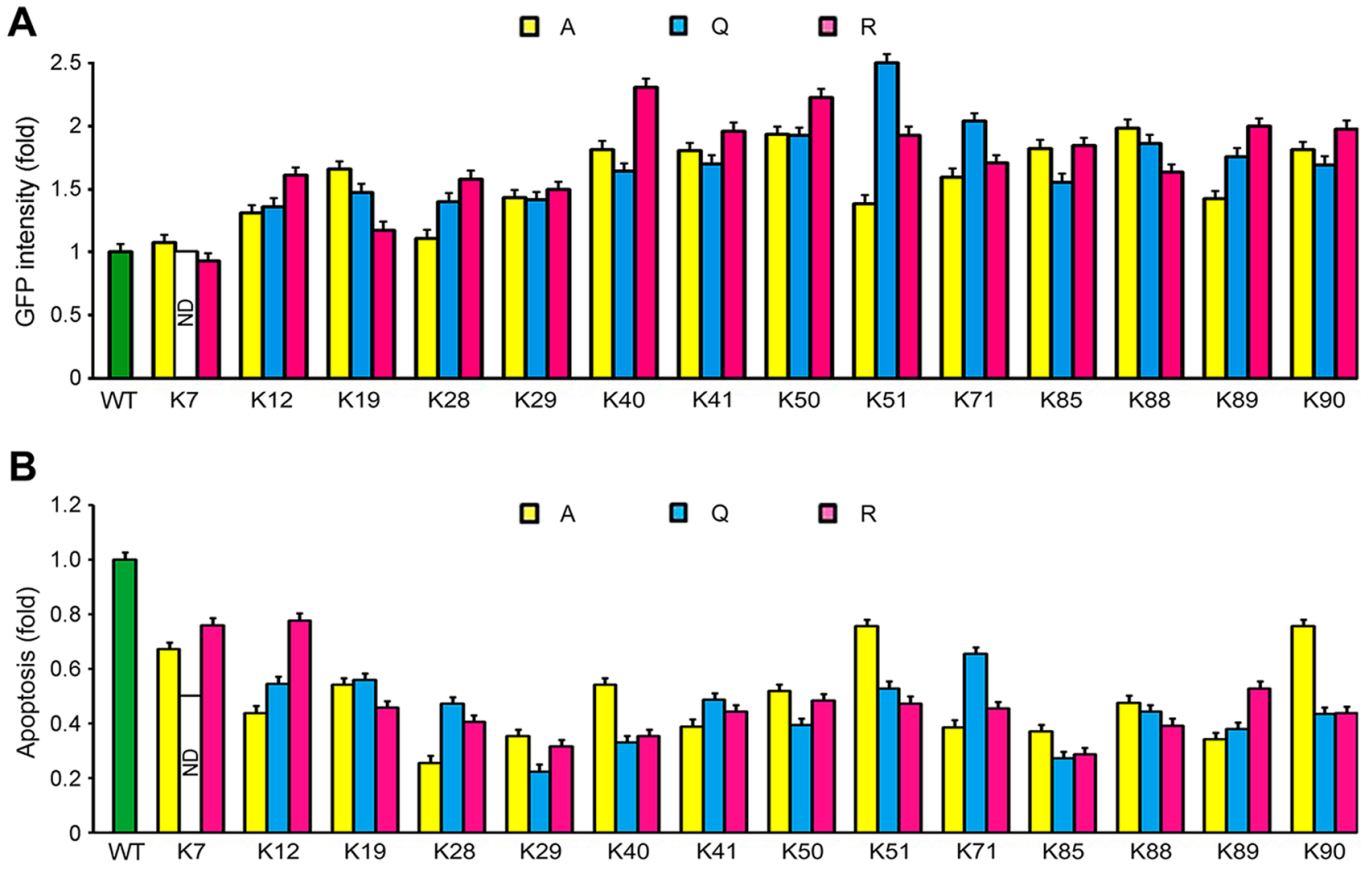
Effects of lysine mutations on Tat-induced apoptosis in Jurkat cells. (A) Jurkat cells were transfected with various GFP-Tat plasmids or the empty vector for 96 hours, and the fluorescence intensity of GFP-tagged proteins was examined by flow cytometry. (B) Experiments were performed as in A, and the percentage of apoptotic cells was analyzed by nuclear staining and normalized to GFP intensity and then to the wild-type control. WT, wild-type; ND, not determined.

We then investigated the effects of lysine mutations on Tat-induced apoptosis in Jurkat T cells. The lysine mutants of Tat were expressed well in Jurkat cells (Fig. 7A). Interestingly, the K7R, K12R, K51A, and K90A mutations induced more robust apoptosis compared with other lysine mutants; however, all the lysine mutants examined compromised to different agrees the ability of Tat to induce apoptosis in Jurkat cells (Fig. 7B).

### Lysine Mutations Regulate the Binding of Tat to Bromodomain Proteins

**Figure 8:**
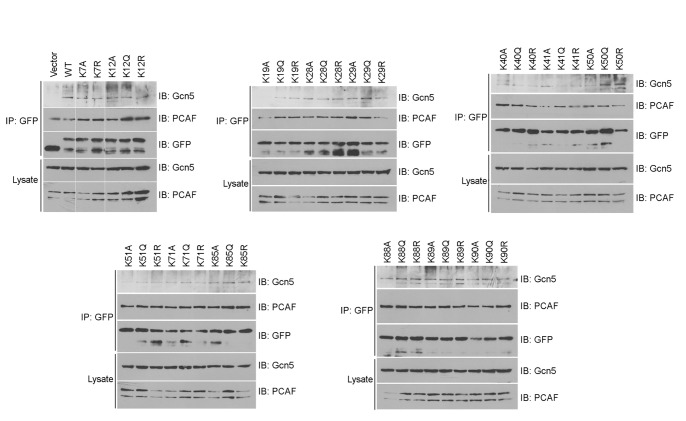
Lysine mutations regulate the binding of Tat to bromodomain proteins. 293T cells were transfected with different GFP-Tat plasmids or the empty vector. Anti-GFP immunoprecipitates and cell lysates were then examined by immunoblotting with anti-Gcn5, anti-PCAF, and anti-GFP antibodies.

Tat acetylation has been shown to modulate its binding to bromodomain proteins, such as Gcn5 and PCAF [23], [24]. Therefore, we studied the effects of lysine mutations on the interaction of Tat with these two proteins. By immunoprecipitation and immublotting, we found that the K19A, K41Q, and K51A mutations decreased Tat binding to Gcn5, whereas the K50Q and K50R mutations increased the binding (Fig. 8). In addition, we found that the K19A, K29R, and K41A mutations decreased Tat binding to PCAF, whereas the K12Q and K12R mutations increased the binding (Fig. 8). These results indicate that lysine mutations may have different effects on the interaction of Tat with bromodomain proteins.

## Discussion

Lysine acetylation, which leads to the formation of a neutral and hydrophobic side chain, is able to modulate diverse protein-protein interactions [27], [28]. Substitution of lysine with glutamine is usually used to mimic lysine acetylation, whereas substitution with alanine can block acetylation although it can neutralize electric charge. In addition, substitution with arginine can block acetylation and preserve electric charge [27], [28]. In the present study, we mutated all the 14 lysine residues in Tat individually to alanine, glutamine, and arginine. Through systematic analysis of the effects of the lysine mutations on Tat subcellular localization and its activities towards HIV-1 transcription, microtubule assembly, mitotic progression, apoptosis, and binding to bromodomain proteins, we have identified several interesting features of Tat.

Our data reveal that lysine mutations modulate the subcellular localization of Tat. The first 48 amino acids of Tat, which contain an acidic region followed by a cysteine-rich region, function as the activation domain. Our data show that mutations in K7 and K41, but not in K12, K19, K28, K29, or K40, affect the localization of Tat. The K90R mutation strikingly compromises the nuclear localization of Tat, indicating that a relatively small side chain or charge interaction may be required for this site and that the K90R mutation may disrupt the binding of Tat with nuclear import factors [29], [30]. The K41Q mutation, which mimics acetylation, greatly increases the nuclear localization of Tat, yet the K41R mutation, which mimics the electric charge of lysine but blocks acetylation, decreases Tat nuclear localization, suggesting that K41 acetylation plays a critical role in the regulation of Tat subcellular localization.

The region of amino acids 49–72 of Tat contains a nuclear localization signal (NLS). Especially, Tat nuclear import is mediated by importin-β, a soluble import factor that selectively binds the NLS of Tat [29]. Human I-mfa domain-containing protein (HIC) is responsible for the cytoplasmic accumulation of Tat due to its interaction with the NLS [31]. In the NLS region of Tat, there are three lysine residues (K50, K51, and K71). For K50, none of mutations obviously affects the nuclear localization of Tat. For K51, the change of lysine to glutamine (K51Q) may mask the NLS of Tat and consequently interfere with its molecular recognition by importin-β or HIC, hence modulating its nuclear localization. Yet, the neutralization mutation K51A and the charge mimicking mutation K51R have little influence on Tat localization, suggesting that K51-assocaited stereo-hindrance instead of acetylation is the major factor to control Tat localization. For K71, the two neutralization mutations K71A and K71Q do not affect Tat localization, but the charge mimicking mutation K71R increases its nuclear localization, suggesting electric charge as the major factor determining K71-associated Tat localization.

Our study also shows that Tat transactivation activity is exquisitely regulated by lysine acetylation. Mutations that alter the amino-terminal 13 amino acids of Tat were originally found to affect Tat transactivation [32]; however, results from a later study conflicted with this interpretation [33]. Our data show that K7 in this region is critical for the modulation of Tat transactivation, as evidenced by the dramatic effect of the K7R mutation. Interestingly, the K28R mutation greatly reduces Tat transactivation activity. K28 has been shown to be important for the stability of the Tat-Cyclin T1-TAR complex [18]. It is possible that the K28R mutation decreases the ability of Tat to interact with Cyclin T1. Our results reveal that the K41A and K41Q mutations remarkably inhibits Tat transactivation, whereas K41R largely preserves the activity, indicating that electric charge of K41 instead of its acetylation is crucial for Tat transactivation activity.

The TAR binding region of Tat (amino acids 49–72) harbors three lysine residues (K50, K51, and K71). Tat is acetylated at K50 by p300 and Gcn5, and this acetylation disrupts the Tat-Cyclin T1-TAR complex, whereas its deacetylation allows Tat to be recycled to TAR for subsequent rounds of HIV-1 transcription [16], [19], [24]. In agreement with these findings, our data reveal that the K50R mutation enhances the transactivation activity of Tat. In contrast, mutations in K51 or K71 have no strong influence on Tat transactivation activity, indicating that although the region of amino acids 49–72 is important for TAR binding, individual amino acid changes do not significantly affect Tat transactivation activity. There are 4 lysine residues (K85, K88, K89, and K90) in the region of amino acids 73–101. Our results show that mutations in K85 and K90 have more evident effect on Tat transactivation activity than K88 and K89; substitution of K85 with glutamine or arginine greatly reduces its activity, whereas substitution of K90 with glutamine or arginine increases its activity.

Tat is a tubulin-binding protein and can promote tubulin polymerization into microtubules, causing the formation of abnormally stable microtubules [6], [7]. Our results reveal that Tat lysine mutations have distinct effects on microtubule assembly. In particular, the K29A, K50R, and K51R mutations reduce the percentage of soluble tubulin, but the majority of other mutations increase the percentage of soluble tubulin. These results are slightly different from those reported previously [6], [7]. Given that the 72-amino acid Tat protein was used in the previous studies, whereas in the present study the 101-amino acid Tat was used, it is possible that the difference in the length of Tat may lead to different three-dimensional structures, which in turn may affect the interaction of Tat with tubulin and its modulation of microtubule assembly.

Microtubule dynamics are known to take an important part in the progression of mitosis. Suppression of microtubule dynamics by diverse chemical agents have been reported to cause mitotic arrest, ultimately resulting in apoptosis [34]. Interestingly, by lysine mutation analysis, we find that the effects of Tat on mitotic progression and apoptosis are not entirely attributed to its activity towards microtubule assembly. Our results indicate that the modulation of mitotic progression and apoptosis by Tat and its regulation of microtubule assembly may involve different mechanisms. It is possible that lysine mutations may modulate the interaction of Tat with tubulin as well as other proteins. In addition, although the effects of Tat on mitotic progression, apoptosis, and microtubule assembly are not generally related, mutations in several residues (e.g. K7, K41, and K88) do show some correlation. As Tat is a very flexible protein, our results suggest that Tat may regulate diverse cellular activities through binding to different proteins and that the acetylation of distinct lysine residues in Tat may modulate its interaction with various partners.
